# Alterations to mTORC1 signaling in the skeletal muscle differentially affect whole-body metabolism

**DOI:** 10.1186/s13395-016-0084-8

**Published:** 2016-03-21

**Authors:** Maitea Guridi, Barbara Kupr, Klaas Romanino, Shuo Lin, Denis Falcetta, Lionel Tintignac, Markus A. Rüegg

**Affiliations:** Biozentrum, University of Basel, 4056 Basel, Switzerland; Present address: Neuromuscular Research Center, Departments of Neurology and Biomedicine, Pharmazentrum, University of Basel, 4056 Basel, Switzerland

**Keywords:** Muscle, Myopathy, Metabolism, Diabetes, mTOR, TSC1, Raptor

## Abstract

**Background:**

The mammalian target of rapamycin complex 1 (mTORC1) is a central node in a network of signaling pathways controlling cell growth and survival. This multiprotein complex integrates external signals and affects different nutrient pathways in various organs. However, it is not clear how alterations of mTORC1 signaling in skeletal muscle affect whole-body metabolism.

**Results:**

We characterized the metabolic phenotype of young and old raptor muscle knock-out (RAmKO) and TSC1 muscle knock-out (TSCmKO) mice, where mTORC1 activity in skeletal muscle is inhibited or constitutively activated, respectively. Ten-week-old RAmKO mice are lean and insulin resistant with increased energy expenditure, and they are resistant to a high-fat diet (HFD). This correlates with an increased expression of histone deacetylases (HDACs) and a downregulation of genes involved in glucose and fatty acid metabolism. Ten-week-old TSCmKO mice are also lean, glucose intolerant with a decreased activation of protein kinase B (Akt/PKB) targets that regulate glucose transporters in the muscle. The mice are resistant to a HFD and show reduced accumulation of glycogen and lipids in the liver. Both mouse models suffer from a myopathy with age, with reduced fat and lean mass, and both RAmKO and TSCmKO mice develop insulin resistance and increased intramyocellular lipid content.

**Conclusions:**

Our study shows that alterations of mTORC1 signaling in the skeletal muscle differentially affect whole-body metabolism. While both inhibition and constitutive activation of mTORC1 induce leanness and resistance to obesity, changes in the metabolism of muscle and peripheral organs are distinct. These results indicate that a balanced mTORC1 signaling in the muscle is required for proper metabolic homeostasis.

**Electronic supplementary material:**

The online version of this article (doi:10.1186/s13395-016-0084-8) contains supplementary material, which is available to authorized users.

## Background

The highly conserved serine/threonine protein kinase mammalian target of rapamycin (mTOR) is known to control numerous cellular processes related to cell growth [45]. mTOR assembles into two functionally distinct multiprotein complexes, the rapamycin-sensitive mTOR complex 1 (mTORC1) and mTORC2, which is only sensitive to prolonged rapamycin treatment [33]. mTORC1 is a central sensor of growth factors and nutrients in various cell types and has been described to play an important role in different pathologies like cancer, metabolic diseases, and aging [21]. Because of its central role in metabolism, the mTOR pathway is extensively studied for its function in type 2 diabetes [29]. mTORC1 is also highly active in the liver and skeletal muscle of obese and high-fat-fed rodents [17, 42]. Inhibition of mTOR signaling by rapamycin prolongs lifespan in several species including mice [15], a treatment that has been proposed to mimic calorie restriction [36]. Paradoxically, prolonged treatment with rapamycin causes glucose intolerance and insulin resistance [10, 13, 16], which has been interpreted to be the result of the inactivation of mTORC2 [20]. In the skeletal muscle, mTORC1 regulates muscle mass by affecting both protein synthesis and degradation [21].

As it is difficult to distinguish the contribution of different tissues on the systemic effects of rapamycin treatment, several laboratories have generated various mouse models with tissue-specific deletions of essential components of the mTORC1 pathway. White adipose tissue (WAT)-specific deletion of *rptor* (gene coding for raptor), which is essential for the activity of mTORC1, leads to improved insulin sensitivity and reduced adipocyte number and size [27]. Inactivation of mTORC1 in the liver leads to resistance to hepatic steatosis and hypercholesteremia induced by a Western diet [26]. While those tissues are the primary sites controlling metabolism, the skeletal muscle has also been shown to contribute to whole-body metabolism. For example, the skeletal muscle is the major site of glucose uptake in response to food intake and insulin and thus can contribute to type 2 diabetes [11]. Accordingly, patients with muscular dystrophies often develop metabolic complications like glucose intolerance and insulin resistance [30, 34]. Similarly, sustained activation of mTORC1 leads to metabolic changes at the whole-body level [14].

In this study, we compared as to how activation or inactivation of mTORC1 in the skeletal muscle affect systemic energy homeostasis. We show that both fatty acid and glucose metabolism are dependent on proper mTORC1 signaling. In mice with muscle-specific depletion of raptor (i.e., inactive mTORC1), the metabolic changes correlate with the upregulation of class II histone deacetylases (HDACs). On the contrary, muscle-specific depletion of tuberous sclerosis complex 1 (TSC1) (i.e., constant activation of mTORC1) leads to an upregulation of transcripts involved in glucose and fatty acid metabolism in various metabolic organs [14] but causes glucose intolerance and late-onset damage in both the liver and the kidneys. These data thus provide evidence that mTORC1 signaling in the skeletal muscle is a major regulator of whole-body metabolism and they suggest that muscle mTORC1 could be a valuable target for the treatment of metabolic complications associated with muscle diseases including muscular dystrophies.

## Material and methods

### Animal experiments

Generation of TSC1 muscle knock-out (TSCmKO) and raptor muscle knock-out (RAmKO) mice and their genotyping were described before [5, 7, 18]. Control mice were littermates floxed for *Rptor* (gene encoding raptor) or *Tsc1* but not expressing Cre recombinase. TSCmKO and RAmKO mice were always compared to a control group of littermates. Initial statistical analysis was always done separately using the respective controls. At the young age, control mice for RAmKO and TSCmKO mice were pooled as those mice had the same age and because statistics was not altered when experimental groups were compared to non-pooled controls. All data shown represent new cohorts of mice although some of the metabolic phenotype of TSCmKO mice have been published before [14]. The fact that those data are confirmatory is mentioned throughout the text. Mice were maintained in a conventional facility with a fixed light cycle (23 °C—12-h dark-light cycle) and were fed standard chow (KLIBA NAFAG, 1304811) or a high-fat diet (HFD) containing 60 % fat (KLIBA NAFAG, 2127.PH.A05) ad libitum. HFD was started at 8 or 10 weeks of age, respectively, for RAmKO and TSCmKO mice and continued for 12 weeks. Body composition was determined by magnetic resonance with the EchoMRI-100H body composition analyzer (EchoMRI™) in immobilized conscious mice. In some experiments, mice were intraperitoneally injected with insulin at 2 p.m. (0.75 U/kg, Humalog, Eli Lilly) after a 5-h fast and euthanized 45 min after for tissue collection. Euthanasia in the rest of the mice was performed at 10 a.m. after food removal at 6 a.m. of the same morning. Both male and female mice were used for this study after confirming that the phenotype observed was not dependent on gender. Data from male mice are shown in the main figures, whereas results from female mice, when available, are shown in the Additional files. All procedures were performed in accordance with the Swiss regulations for animal experimentation and approved by the veterinary commission of the Canton Basel-Stadt.

### Metabolic measurements

Glucose, lactate, and insulin plasma levels were analyzed in tail vein blood after a 4-h fast (6 a.m. to 10 a.m.) with One Touch Ultra Easy glucose meter (LifeScan, Inc.), Lactate Pro test strips (Arkray Factory, Inc.), and Ultra-Sensitive Mouse Insulin ELISA kit (Crystal Chem, Inc.), respectively. ATP content and glycogen amount in the muscle and liver were determined by using a luminescence assay (CellTiter-Glo Luminescent Cell Viability Assay, Promega) and a Glycogen assay kit (Sigma-Aldrich), respectively. A full analysis of plasma parameters was performed with a cobas C 111 machine (Roche) after a 4-h fast (6 a.m. to 10 a.m.).

### Indirect calorimetry by Comprehensive Lab Animal Monitoring System, Columbus Instruments (CLAMS)

Mice were acclimatized for 2 days (individual housing) followed by data acquisition over 3 to 4 days. Activity (i.e., ambulatory movement determined by laser counts in *X* and *Y* coordinates), feeding, and drinking behaviors were measured daily over a period of 4 days. Oxygen use and carbon dioxide production was measured, and energy expenditure was calculated with the Weir equation. Respiratory exchange ratio (RER) was calculated as carbon dioxide volume (VCO_2_)/oxygen volume (VO_2_). Data were normalized to body weight.

### Intra-peritoneal (IP) insulin tolerance test (ITT) and glucose tolerance test (GTT)

After an overnight starvation for GTT and a 5-h fast for ITT (from 9 a.m. to 2 p.m.), mice were intraperitoneally injected with 1.5 g/kg glucose (Merck) or 0.75 U/kg insulin (Humalog, Eli Lilly), respectively. Basal blood glucose was measured before the injection from tail vein blood and at the indicated time points after the intraperitoneal injection.

### Histology

The liver and *tibialis anterior* (TA) muscle, frozen in liquid nitrogen-cooled isopentane, were cut into 10-μm-thick cross sections. Sections were stained with hematoxylin (Merck)-eosin (Sigma-Aldrich) and Oil Red-O (Sigma-Aldrich) and mounted with glycerol gelatin (Sigma-Aldrich).

### Quantitative real-time PCR

Total RNA from RAmKO and control mice was isolated (SV Total RNA isolation System, Promega), and equal amounts of RNA were reverse transcribed using a mixture of oligodT and random hexamer primers (iScript cDNA Synthesis Kit, Bio-Rad). Quantitative real-time PCR was performed using SYBR Green (Power SYBR Green Master Mix, Applied Biosystems) and StepOne™ Software 2.1. (Applied Biosystems). Expression levels for each gene of interest were normalized to the mean cycle number using real-time PCR for the housekeeping gene-encoding β-actin, whose expression was not altered between RAmKO and control mice (Additional file 1: Figure S1A). All experiments were performed in triplicates. Primers used are listed in Additional file 2: Table S1.

### Western blotting

Proteins were extracted from the TA muscle, liver, WAT, and BAT as described previously [5]. Total protein levels were determined using a reducing agent-compatible BCA Protein Assay (Pierce). Signal was captured on a Fusion Fx machine (Vilber Lourmat); gray values were corrected for background and analyzed with the FUSION Capt FX software. Quantification of each protein was normalized to the loading control (α-actinin or β-actin). To determine the extent of protein phosphorylation, relative intensity of the band using a phospho-specific antibody was divided by the amount of protein as determined by a pan-specific antibody. Samples from the four groups of mice were all run together on the same gel, and quantification was done relative to the values of the control group for each genotype. Antibodies are listed in Additional file 1: Figure S1B.

### Statistical analyses

Compiled data are expressed as mean ± SEM and *n* (total number of knock-out mice). Measurements were performed at least in three independent sets of experiments. Statistical comparison of two conditions was performed using the Student’s *t* test; comparison of three or more groups was performed using the one-way or two-way ANOVA test with Tukey’s correction for multiple comparisons, and data where time was a variable were analyzed by linear regression (GraphPad Prism Software). A 0.05 level of confidence was accepted for statistical significance.

## Results

### Modification of skeletal muscle mTORC1 signaling affects whole-body metabolism

We have previously reported that inhibition of mTORC1 activity in the skeletal muscle by raptor depletion (RAmKO mice) results in a lethal myopathy [5]. Interestingly, sustained activation of mTORC1 by depletion of TSC1 (TSCmKO mice) also results in a late-onset myopathy [7]. In addition, RAmKO mice show alterations in glucose metabolism in the muscle [5], whereas TSCmKO show strong changes in their fatty acid metabolism at the whole-body level [14]. As the skeletal muscle phenotypes of RAmKO and TSCmKO converge at older age, we decided to also perform a detailed characterization and comparison of RAmKO and TSCmKO mice at the whole-body level.

**Fig. 1:**
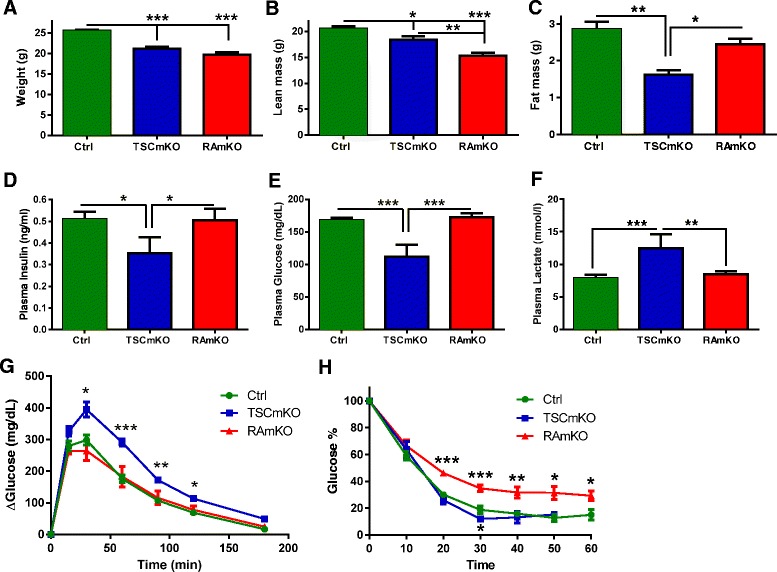
Alterations of mTORC1 signaling in the skeletal muscle affects whole-body metabolism. **a** Body weight is lower in TSCmKO (*n* = 10) and RAmKO (*n* = 17) mice at 10 weeks of age when compared to control (*Ctrl*) mice (*n* = 14). **b**–**c** Lean mass (**b**) is lower in TSCmKO (*n* = 13) and RAmKO (*n* = 16) mice while fat mass (**c**) is only decreased in TSCmKO mice (*n* = 6) when compared to control (*Ctrl*) mice at 10 weeks of age (*n* = 21). **d**–**e** Plasma insulin levels (**d**) and glucose levels (**e**) are decreased in 10-week-old TSCmKO mice (*n* = 12) while they are unchanged in 10-week-old RAmKO mice (*n* = 6) when compared to control (*Ctrl*) mice (*n* = 10). **f** Plasma lactate levels are increased in 10-week-old TSCmKO mice (*n* = 6) while they are unchanged in 10-week-old RAmKO mice (*n* = 6) when compared to control (*Ctrl*) mice (*n* = 12). **g**–**h** TSCmKO (*n* = 6) but not RAmKO mice (*n* = 6) show glucose intolerance in a GTT (**g**) while RAmKO (*n* = 6) but not TSCmKO mice (*n* = 6) show insulin resistance in an ITT (**h**) at 10 weeks of age when compared to control (*Ctrl*) mice (*n* = 10). Data presented are all from male mice of the indicated genotypes. Data represent mean ± SEM. *p < 0.05, **p < 0.01, ***p < 0.001

First, we analyzed the body composition by EchoMRI using 10-week-old mice, an age at which neither of the mice show myopathic signs [4, 5, 7, 14]. Both male and female TSCmKO mice were significantly lighter when compared to age-matched control mice (Fig. 1a; Additional file 1: Figure S2A). In RAmKO mice, male mice were also lighter than their control littermates (Fig. 1a) while this difference did not reach significance in females (Additional file 1: Figure S2A). This difference in the young RAmKO mice was due to a lower lean mass without affecting the amount of fat (Fig. 1b, c; Additional file 1: Figure S2B and S2C). In young TSCmKO mice, lean mass was moderately changed whereas the amount of fat was strongly reduced (Fig. 1b, c; Additional file 1: Figure S2B and S2C). RAmKO mice showed no changes in insulin (Fig. 1d) and plasma glucose levels (Fig. 1e; Additional file 1: Figure S2D), whereas those plasma parameters were lower in TSCmKO mice as previously reported [14] and now confirmed in a new set of mice (Fig. 1d, e; Additional file 1: Figure S2D). Besides the changes in blood glucose and insulin, the concentration of lactate was also increased in TSCmKO mice but not in RAmKO mice (Fig. 1f). As those plasma profiles suggest changes in the glucose uptake capacity, we next performed glucose and insulin tolerance tests. They revealed that TSCmKO mice were glucose intolerant (Fig. 1g) and slightly more sensitive to insulin (Fig. 1h). RAmKO mice had the reciprocal phenotype with normal glucose tolerance (Fig. 1g) but insulin resistance (Fig. 1h). A similar reciprocal phenotype was observed for the basal metabolism as energy expenditure was increased in 10-week-old RAmKO mice but not in TSCmKO mice (Table 1). Thus, these results show that some of the early changes in the whole-body metabolism are differentially affected in RAmKO and TSCmKO mice.

**Table 1:** CLAMS analysis of 10-week-old TSCmKO and RAmKO mice

	Ctrl	TSCmKO	*P*	Ctrl	RAmKO	*P*
Drink (ml/g/day)	0.31 ± 0.04	0.34 ± 0.06	NS	0.28 ± 0.04	0.30 ± 0.10	NS
Feed (g/g/day)	0.58 ± 0.17	0.58 ± 0.05	NS	0.42 ± 0.06	0.60 ± 0.14	*
CO_2_ (l/g/day)	0.37 ± 0.02	0.36 ± 0.03	NS	0.34 ± 0.04	0.41 ± 0.03	*
O_2_ (l/g/day)	0.39 ± 0.02	0.38 ± 0.02	NS	0.37 ± 0.05	0.44 ± 0.04	*
RER	0.94 ± 0.01	0.93 ± 0.03	NS	0.90 ± 0.01	0.91 ± 0.01	NS
Heat (kcal/h/g)	0.020 ± 0.00	0.019 ± 0.00	NS	0.026 ± 0.00	0.032 ± 0.00	*
X-amb (counts/h)	1078.9 ± 230.2	938.66 ± 314.8	NS	935.3 ± 357.3	938.7 ± 314.8	NS
Y-amb (counts/h)	187.9 ± 45.8	186.1 ± 51.6	NS	169.50 ± 93.17	140.19 ± 61.47	NS

Student’s *t* test. Values represent mean ± SEM over a period of 3 days. Data presented are of male mice. X-amb and Y-amb refer to ambulatory movement measured by laser counts in *X* and *Y* coordinates

*NS* not significant

*p < 0.05 (*n* = 6)

### TSCmKO and RAmKO mice are both resistant to a high-fat diet

**Fig. 2:**
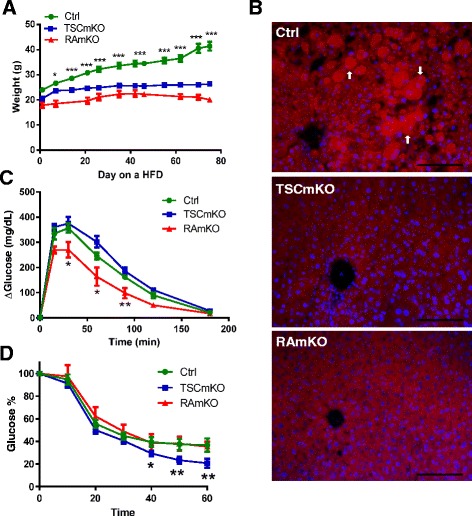
TSCmKO and RAmKO mice are both resistant to a high-fat diet. **a** TSCmKO (*n* = 7) and RAmKO (*n* = 5) mice do not gain significant weight on a HFD when compared to control (*Ctrl*) mice (*n* = 12). **b** TSCmKO and RAmKO mice are resistant to HFD-induced hepatic steatosis, shown by decreased lipid accumulation in Oil Red O-stained liver (*n* = 3). *Arrows* indicate Oil Red O-stained lipids. Scale bar: 100 μm. **c** Twenty-week-old RAmKO mice (*n* = 5) show an increased glucose tolerance on a GTT after a HFD when compared to TSCmKO (*n* = 6) and control (*Ctrl*) mice (*n* = 12). **d** Twenty-week-old TSCmKO mice (*n* = 6) show increased insulin sensitivity on an ITT after a HFD when compared to RAmKO (*n* = 5) and control (*Ctrl*) mice (*n* = 12). Data presented are all from male mice of the indicated genotypes. Data represent mean ± SEM. *p < 0.05, **p < 0.01, ***p < 0.001

To test how the mice perform under metabolic stress, we fed both RAmKO and TSCmKO mice a HFD for 12 weeks, starting at the age of 8 or 10 weeks, respectively. Neither TSCmKO nor RAmKO mice gained as much weight as the control mice (Fig. 2a). RAmKO mice maintained significantly lower fat and lean mass while on a HFD (Additional file 1: Figure S2E and S2F) as did TSCmKO mice [14]. Control mice also developed hepatic steatosis whereas TSCmKO and RAmKO were resistant (Fig. 2b). Prolonged HFD feeding causes type 2 diabetes [43]. Consistent with the HFD resistance, plasma glucose levels were reduced in TSCmKO [14] and RAmKO mice (Additional file 1: Figure S2G). In addition, RAmKO mice showed an improved glucose tolerance compared to TSCmKO and control mice under the HFD (Fig. 2c). In contrast, TSCmKO showed an increased insulin sensitivity during the HFD when compared to RAmKO and control mice (Fig. 2d) [14]. As previously shown [14], TSCmKO mice placed on HFD ate and drank more, showed increased activity and energy expenditure, but decreased respiratory exchange ratio (Table 2). On the other hand, RAmKO mice showed a decrease in their activity and the respiratory exchange ratio (Table 2), indicating a preference for fatty acid metabolism as a source of energy [12]. These results show that both activation and inhibition of mTORC1 in the skeletal muscle conferred resistance to a HFD and they indicate that different mechanisms underlie this phenotype.

**Table 2:** CLAMS analysis of TSCmKO and RAmKO mice on a HFD

	Ctrl	TSCmKO	*P*	Ctrl	RAmKO	*P*
Drink (ml/g/day)	0.11 ± 0.04	0.16 ± 0.02	**	0.15 ± 0.03	0.15 ± 0.06	NS
Feed (g/g/day)	0.21 ± 0.06	0.36 ± 0.12	*	0.27 ± 0.02	0.29 ± 0.13	NS
CO_2_ (l/g/day)	0.24 ± 0.03	0.32 ± 0.02	***	0.29 ± 0.04	0.30 ± 0.04	NS
O_2_ (l/g/day)	0.31 ± 0.04	0.41 ± 0.02	**	0.36 ± 0.04	0.40 ± 0.06	NS
RER	0.75 ± 0.01	0.77 ± 0.01	**	0.79 ± 0.01	0.75 ± 0.01	**
Heat (kcal/h/g)	0.021 ± 0.00	0.028 ± 0.00	**	0.019 ± 0.00	0.020 ± 0.00	NS
X-amb (counts/h)	557.5 ± 122.4	616.9 ± 80.6	NS	601.2 ± 103.8	294.7 ± 71.1	**
Y-amb (counts/h)	55.5 ± 7.8	79.1 ± 25.1	0.05	49.4 ± 10.5	42.3 ± 15.9	NS

Student’s *t* test. Values represent mean ± SEM over a period of 4 days. Data presented are of male mice. X-amb and Y-amb refer to ambulatory movement measured by laser counts in *X* and *Y* coordinates

*NS* not significant

*p < 0.05

**p < 0.01

***p < 0.001 (*n* = 6)

### TSCmKO but not RAmKO mice show changes in other metabolic organs

**Fig. 3:**
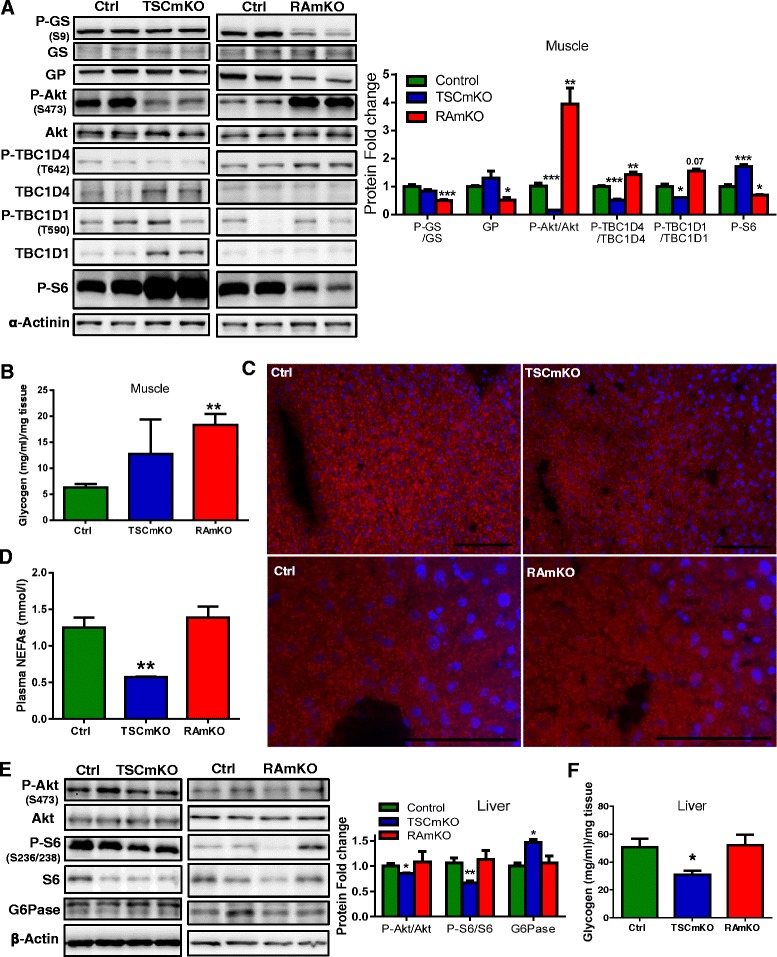
TSCmKO but not RAmKO mice show changes in non-targeted metabolic organs. **a** Immunoblots of TA muscle from 10-week-old TSCmKO, RAmKO, and control (*Ctrl*) mice are shown for the indicated phospho (P)- and total proteins (*n* = 4). Protein expression is normalized to α-actinin. Quantification of phosphorylation is shown relative to the total amount of each protein except for P-S6. **b** Glycogen amount is increased in the *gastrocnemius* muscle of 12-week-old RAmKO mice (*n* = 5) compared to TSCmKO (*n* = 7) and control (*Ctrl*) mice (*n* = 13). **c** Liver lipid content is decreased in 12-week-old TSCmKO as shown by Oil Red O staining, while it is unchanged in 10-week-old RAmKO mice (*n* = 3). Scale bar: 100 μm. **d** The concentration of non-sterified fatty acids in the plasma is decreased in 10-week-old TSCmKO mice (*n* = 4) compared to RAmKO (*n* = 6) and control (*Ctrl*) mice (*n* = 8). **e** Immunoblots of the liver from 10-week-old TSCmKO, RAmKO, and control (*Ctrl*) mice are shown for the indicated phospho (P)- and total proteins (*n* = 4). Protein expression is normalized to β-actin. Quantification of the phospho-protein is shown relative to the amount of each protein. **f** Glycogen amount is decreased in the liver from 12-week-old TSCmKO mice (*n* = 4) compared to RAmKO (*n* = 3) and control (*Ctrl*) mice (*n* = 6). Data presented are all from male mice of the indicated genotypes. Data represent mean ± SEM. *p < 0.05, **p < 0.01, ***p < 0.001

The activation state of the serine/threonine kinase protein kinase B (Akt/PKB) is altered in RAmKO and TSCmKO mice because of the negative feedback loop from S6K on IRS1 [42]. Thus, in RAmKO mice, lack of activation of S6K causes increased phosphorylation of Akt/PKB [5], whereas Akt/PKB phosphorylation is dampened in TSCmKO mice [4]. As Akt/PKB signaling is an important regulator of carbohydrate metabolism [35], we next examined Akt/PKB targets involved in glucose absorption and storage. As previously shown [5, 14, 31], we confirmed that glycogen phosphorylase levels were decreased in RAmKO muscle, as well as glycogen synthase phosphorylation (Fig. 3a) while they were unchanged in TSCmKO muscle. Moreover, phosphorylation of the Akt/PKB substrate of 160 kDa (AS160/TBC1D4), responsible for glucose transporter 4 (GLUT4) translocation to the sarcolemma upon insulin stimulation [6], was decreased in TSCmKO when measured relative to the amount of TBC1D4. Similarly, phosphorylation of TBC1D1, another Akt/PKB substrate involved in basal glucose absorption into the skeletal muscle [6], was also reduced in TSCmKO muscle relative to the total amount of TBC1D1 (Fig. 3a). Interestingly, both TBC1D4 and TBC1D1 protein levels were increased in TSCmKO muscle (Fig. 3a). In contrast, phosphorylation of TBC1D4 was increased in RAmKO muscle and there was a trend for increased TBC1D1 phosphorylation (Fig. 3a). Overall, the observed changes correlated well with the increased glycogen levels (Fig. 3b and Additional file 1: Figure S3A). Thus, modifications of the mTORC1 activity in the skeletal muscle lead to dysregulated Akt/PKB signaling that result in changes in glucose transport and storage.

We have previously reported that TSCmKO mice show browning of white adipose tissue and increased fatty acid oxidation in the liver [14]. In contrast to the decrease in lipids in TSCmKO mice (Fig. 3c), lipid content seemed unchanged in RAmKO liver (Fig. 3c). In agreement with this, plasma non-esterified fatty acids were decreased in TSCmKO mice but were the same as in the controls or RAmKO mice (Fig. 3d). We decided to analyze Akt/PKB signaling in the liver because insulin is a key regulator of gluconeogenesis and glycogenolysis in this organ. While Akt/PKB and mTORC1 activities were unchanged in RAmKO mice, both were downregulated in the liver of TSCmKO mice (Fig. 3e). This is likely a consequence of the decreased plasma insulin levels of the TSCmKO mice and not lack of responsiveness, as the liver from TSCmKO mice responded to insulin-like controls (Additional file 1: Figure S3B). Moreover, the amount of glucose-6 phosphatase was higher in TSCmKO mice than in the controls or RAmKO mice (Fig. 3e). In correlation with these protein changes, the glycogen amount was reduced in the livers from TSCmKO but not from RAmKO mice (Fig. 3f). In addition, none of the genes that were reported to be changed in TSCmKO mice involved in fatty acid or glucose metabolism in the liver; white adipose tissue or brown fat of TSCmKO mice [14] were changed in RAmKO mice (Additional file 1. Liver: Figure S3C, S3E, and S3F; white adipose tissue: Figure S3C; brown fat: Figure S3D). These results show that under a normal diet, mTORC1 activation in the skeletal muscle causes changes in other metabolic organs, such as the liver and adipose tissue. In contrast, the effect of its inhibition is limited to the targeted skeletal muscle.

### Strong downregulation of metabolic genes and increased levels of HDACs in RAmKO skeletal muscle

**Fig. 4:**
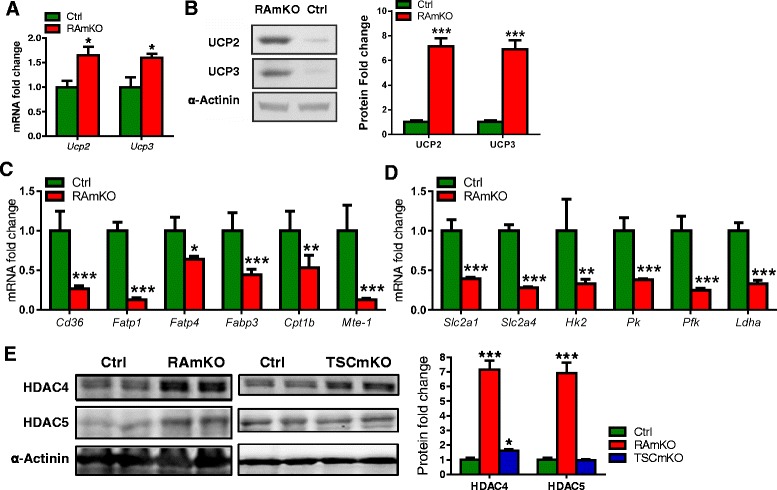
Strong downregulation of metabolic genes and increased levels of HDACs in RAmKO skeletal muscle. **a**–**b** Increased expression (**a**) and protein levels (**b**) of UCP2 and UCP3 in the skeletal muscle of 12-week-old RAmKO mice (*n* = 4) when compared to control (*Ctrl*) littermates (*n* = 4). **c**–**d** Genes involved in fatty acid (**c**) and glucose metabolism (**d**) are expressed at lower levels in the skeletal muscle of 12-week-old RAmKO mice (*n* = 4) when compared to control (*Ctrl*) littermates (*n* = 4). **e** HDAC4 protein levels are higher in the TA muscles of RAmKO (*n* = 4) and TSCmKO (*n* = 4) at 12 weeks of age. In contrast, the levels of HDAC5 are only higher in RAmKO (*n* = 4) as shown by immunoblot. Note: both bands of the HDAC proteins were included in the quantification. Protein expression is normalized to α-actinin. Data presented are all from male mice of the indicated genotypes. Data represent mean ± SEM. *p < 0.05, **p < 0.01, ***p < 0.001

Uncoupling proteins (UCPs) uncouple the proton gradient in the inner membrane of the mitochondria thereby regulating efficiency of ATP production and energy expenditure in cells [1]. In WAT, mTORC1 regulates UCP expression [27]. Thus, we determined mRNA and protein levels of UCP in the skeletal muscle. Both mRNA and protein abundance of UCP2 and of the muscle-specific UCP3 were significantly increased in RAmKO mice (Fig. 4a, b). Whereas ATP levels in TSCmKO mice are reduced, whose muscles also contain a higher amount of UCP2 [14], the ATP content in the muscle of RAmKO mice was identical to controls (Additional file 1: Figure S4). Besides the changes in UCPs, expression of genes involved in fatty acid transport and oxidation, like *Fatp4*, *Fabp3*, or *Cpt1b*, was decreased in the muscle of young RAmKO mice compared to control littermates (Fig. 4c). In addition, expression of glucose transporters and genes involved in glycolysis was also reduced in RAmKO skeletal muscle (Fig. 4d), as opposed to the increased expression of genes involved in glucose absorption and fatty acid oxidation seen in TSCmKO muscle [14]. To better understand the possible pathways involved in the regulation of those metabolic genes, we next analyzed the expression of class II histone deacetylases 4 and 5 (HDAC4 and HDAC5), which are known to regulate glycolytic proteins [23, 37]. HDAC4 and HDAC5 protein levels were increased in RAmKO mice, while there was only a slight, but significant increase of HDAC4 in TSCmKO muscle (Fig. 4e). Thus, the strong increase in HDAC4 and HDAC5 in RAmKO skeletal muscle could contribute to the decreased expression of genes involved in fatty acid and glucose metabolism.

### Myopathy pre-dominates the metabolic changes in the two animal models at older age

**Fig. 5:**
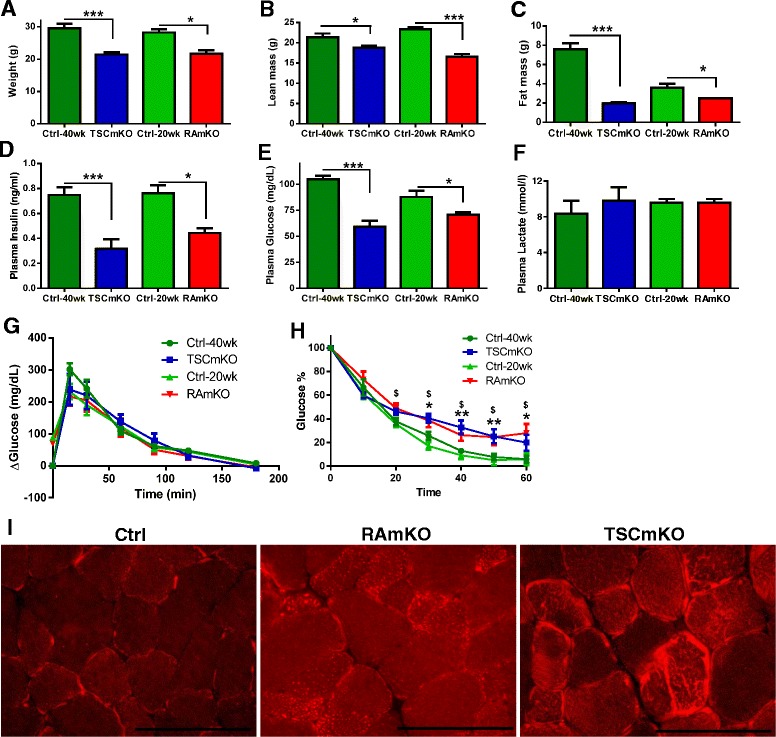
Myopathy pre-dominates the metabolic changes at higher age. **a** Body weight is decreased in both TSCmKO (*n* = 8) and RAmKO (*n* = 4) mice at 40 and 20 weeks of age, respectively, when compared to control (*Ctrl*) littermates (*n* = 9). **b**–**c** Both lean mass (**b**) and fat mass (**c**) are decreased in TSCmKO (*n* = 10) and RAmKO (*n* = 8) mice at 40 and 20 weeks of age, respectively, when compared to control (*Ctrl*) littermates (*n* = 10). **d**–**e** Both plasma insulin (**d**) and glucose (**e**) levels are decreased in TSCmKO (*n* = 8) and RAmKO (*n* = 5) mice at 40 and 20 weeks of age, respectively, when compared to control (*Ctrl*) littermates (*n* = 12). **f** Plasma lactate levels are unchanged in 40-week-old TSCmKO (*n* = 3) and 20-week-old RAmKO (*n* = 3) mice compared to control (*Ctrl*) littermates (*n* = 6). **g**–**h** TSCmKO (*n* = 5) and RAmKO mice (*n* = 5) show normal glucose tolerance (**h**) and develop insulin resistance (**i**) at 40 and 20 weeks of age, respectively, when compared to control (*Ctrl*) littermates (*n* = 10). **i** Oil Red O staining of the *gastrocnemius* muscle in 40-week-old TSCmKO and RAmKO mice indicates increased lipid accumulation (*n* = 3). Scale bar: 100 μm. Data presented are all from male mice of the indicated genotypes. Data represent mean ± SEM. *p < 0.05, **p < 0.01, ***p < 0.001

Previous work has shown that both RAmKO and TSCmKO mice develop a myopathy [5, 7]. To investigate whether the metabolic changes in peripheral organs would also converge at older age, we next compared the overall metabolism between 20-week-old RAmKO and 40-week-old TSCmKO mice, the age at which the myopathy is fully developed [5, 7]. Body weights were significantly reduced in male and female TSCmKO and male RAmKO mice (Fig. 5a; Additional file 1: Figure S5A), which was due to lower lean (Fig. 5b; Additional file 1: Figure S5B) and fat mass (Fig. 5c; Additional file 1: Figure S5C). Basal metabolic analysis revealed an increase in energy expenditure in 40-week-old TSCmKO mice, while in 20-week-old RAmKO mice, the overall energy expenditure was now as in controls (Table 3). Analysis of the blood plasma revealed that insulin (Fig. 5d) and glucose (Fig. 5e) levels were reduced in both TSCmKO and RAmKO mice, while the increased plasma lactate levels of young TSCmKO mice (see Fig. 1f) were normalized in the old mice (Fig. 5f). It is well established that loss of muscle mass also affects glucose metabolism [30, 34]. While glucose tolerance was normal (Fig. 5g), both TSCmKO and RAmKO mice were now insulin resistant (Fig. 5h). Insulin resistance in muscle has been linked to the disruption of lipid dynamics and accumulation of intramyocellular, lipotoxic intermediates [2]. Thus, we analyzed lipid content in the muscle of the mutant mice and found that lipid droplets accumulated in RAmKO muscle and the amount of lipids was increased in TSCmKO muscle (Fig. 5i). These results suggest that the myopathy in RAmKO and TSCmKO mice results in very similar overall perturbation of the metabolism.

**Table 3:** CLAMS analysis of 40-week-old TSCmKO and 20-week-old RAmKO mice

	Ctrl	TSCmKO	*P*	Ctrl	RAmKO	*P*
Drink (ml/g/day)	0.18 ± 0.05	0.22 ± 0.07	NS	0.23 ± 0.11	0.14 ± 0.03	NS
Feed (g/g/day)	0.31 ± 0.09	0.40 ± 0.12	NS	0.37 ± 0.19	0.44 ± 0.13	NS
CO_2_ (l/g/day)	0.28 ± 0.03	0.32 ± 0.04	*	0.25 ± 0.05	0.26 ± 0.02	NS
O_2_ (l/g/day)	0.32 ± 0.03	0.37 ± 0.04	*	0.28 ± 0.05	0.30 ± 0.03	NS
RER	0.85 ± 0.05	0.88 ± 0.05	NS	0.88 ± 0.04	0.87 ± 0.01	NS
Heat (kcal/h/g)	0.022 ± 0.00	0.028 ± 0.00	*	0.026 ± 0.00	0.028 ± 0.00	NS
X-amb (counts/h)	884.1 ± 316.4	578.1 ± 113.1	0.05	820.7 ± 247.7	856.6 ± 434.9	NS
Y-amb (counts/h)	117.1 ± 43.1	75.8 ± 19.5	0.09	100.2 ± 56.4	130.0 ± 48.8	NS

Student’s *t* test. Values represent mean ± SEM over a period of 3 days. Data presented are of male mice. X-amb and Y-amb refer to ambulatory movement measured by laser counts in *X* and *Y* coordinates

*NS* not significant

*p < 0.05 (*n* = 6)

We previously reported that the myopathy of RAmKO mice is particularly severe in the diaphragm, which led us to suggest that respiratory failure might be the cause of death [5]. Low respiration reduces oxygen saturation and causes the accumulation of carbon dioxide in the blood. Blood gas analysis revealed that oxygen and carbon dioxide pressure in the blood was the same as in the controls in 10-week-old RAmKO, but oxygen levels dropped and carbon dioxide increased in 20-week-old, myopathic RAmKO mice (Additional file 2: Table S2). The increase in carbon dioxide also resulted in the lowering of the blood pH indicative of respiratory acidosis (Additional file 2: Table S2). Although we cannot rule out respiratory complications in old, myopathic TSCmKO mice [7], we observed that 40-week-old TSCmKO mice also had polycystic kidneys (Additional file 1: Figure S5D), a frequent cause for acute kidney failure [44]. In agreement with the conclusion that the kidneys were damaged, the amount of creatinine and lactate dehydrogenase (LDH) was significantly elevated in the blood of TSCmKO mice (Table 4). In addition, alanine aminotransferase (ALTL) and aspartate aminotransferase (ASTL) were also increased in the plasma of 40-week-old TSCmKO mice (Table 4), which are commonly used as markers for liver damage [24]. We hypothesize that this kidney damage in old TSCmKO mice was the consequence of prolonged muscle breakdown or rhabdomyolysis [39]. These results suggest that the disease is mainly restricted to the skeletal muscle in the RAmKO mice and thus the mice are likely to die of respiratory failure. In contrast, TSCmKO mice show defects in several tissues and thus they might succumb to diseases in multiple organs, including the skeletal muscle and kidney.

**Table 4:** cobas analysis of 40-week-old TSCmKO and 20-week old RAmKO plasma

Group	ALTL (U/l)	ASTL (U/l)	Uric acid (μmol/l)	Creatinine (μmol/l)	LDH (mmol/l)	TRIGL (mmol/l)	HDL-Chol (mmol/l)	LDL-Chol (mmol/l)	Chol (mmol/l)
Ctrl	40.5 ± 4.3	65.7 ± 10.5	220.8 ± 54.0	10.7 ± 1.7	256.5 ± 76.1	1.1 ± 0.2	2.5 ± 0.5	0.3 ± 0.0	2.7 ± 0.6
TSCmKO	85.4 ± 13.6	308.9 ± 53.3	244.1 ± 60.6	16.6 ± 0.6	567.2 ± 74.2	1.1 ± 0.2	2.26 ± 0.3	0.4 ± 0.0	2.6 ± 0.3
*P*	**	***	NS	***	***	NS	NS	**	NS
Ctrl	31.5 ± 4.7	60.3 ± 14.3	115.3 ± 24.6	16.1 ± 1.7	278.0 ± 99.3	0.7 ± 0.5	2.0 ± 0.3	0.2 ± 0.0	2.3 ± 0.3
RAmKO	38.4 ± 8.7	95.2 ± 12.8	118.8 ± 28.5	15.5 ± 1.9	412.4 ± 58.7	0.5 ± 0.4	1.9 ± 0.2	0.2 ± 0.0	2.3 ± 0.3
*P*	NS	*	NS	NS	NS	NS	NS	NS	NS

Values represent mean ± SEM of male mice. Student’s *t* test

*NS* not significant

*p < 0.05

**p < 0.01

***p < 0.001 (*n* = 5)

## Discussion

The control of energy balance plays a central role in metabolic diseases such as type 2 diabetes and obesity. mTORC1 has been postulated to play an essential role in glucose homeostasis by fine-tuning insulin signaling through Akt/PKB and by controlling metabolic pathways in different tissues [40, 41]. Likewise, mTORC1 has a central role in regulating lipid metabolism and adipogenesis by activating essential transcription factors like peroxisome proliferator-activated receptor gamma (*Pparg*) and sterol regulatory element binding protein 1 (*Srebp1*) [19]. The skeletal muscle is a particularly important player in the regulation of energy balance in the body, serving both as a major glucose and energy-storing tissue, as well as an avid energy consumer during physical activity. mTORC1 also affects muscle mass and integrity by regulating both protein synthesis and degradation, and it has been suggested to be involved in muscle wasting during aging [38]. However, how mTORC1 activity in the skeletal muscle affects whole-body metabolism has not yet been clarified in detail.

Consequences of mTORC1 perturbation have been described in other metabolic tissues, such as WAT or the liver, and revealed differential effects of mTORC1 signaling on tissue and whole-body metabolism, depending on the targeted organs and the mouse models used. For instance, although mTORC1 promotes lipogenesis in the liver cells through sterol regulatory element binding protein (SREBP) activation, this effect was abolished in mice with liver-specific depletion of TSC1 and also resulted in insulin resistance, due to the simultaneous inhibition of Akt/PKB in this tissue [22, 26, 46]. Inactivation of mTORC1 in WAT caused the browning and reduction of fat, resulted in an increase in energy expenditure, and Akt/PKB-dependent insulin sensitivity [28].

**Fig. 6:**
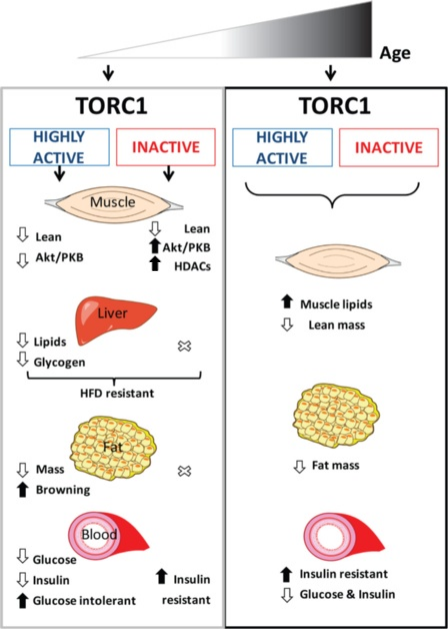
Summary of metabolic changes induced by altered mTORC1 signaling in the skeletal muscle of mice during aging

In our study, we now report on the whole-body consequences of mTORC1 activation and inhibition in the skeletal muscle (Fig. 6). At a young age, mTORC1 inhibition had a stronger effect on the skeletal muscle, causing a significant reduction in lean mass and muscle atrophy [5]. Only few changes were observed at the metabolic level in RAmKO mice, one being increased energy expenditure, most likely a consequence of the higher UCP2 and UCP3 mRNA and protein amount in the muscle. However, RAmKO mice were insulin resistant, which could be a direct consequence of the early muscle atrophy and dysfunctional muscle dynamics. Conversely, at this young age, activation of mTORC1 in the skeletal muscle caused strong changes of the metabolism without yet affecting the structural integrity of the skeletal muscle. We have previously shown that mTORC1 activation induces the release of fibroblast growth factor 21 (FGF21) from the skeletal muscle, which in turn is responsible for several of the metabolic changes, such as hypoglycemia, increased fatty acid oxidation, and reduced body weight [14]. Despite the improved metabolic profile of TSCmKO mice, we now show that they are glucose intolerant. We suggest that this could be a consequence of dampened Akt/PKB signaling and the decreased translocation of glucose transporters to the plasma membrane [6]. Surprisingly, Akt/PKB signaling was also decreased in the liver of TSCmKO, likely due to decreased plasma insulin concentrations. In addition, the glycogen amount in the liver was lower in TSCmKO mice, which correlated with an increase in glucose 6-phosphatase, suggesting increased liver glycogenolysis as compensation for the low plasma glucose concentration. In contrast to the TSCmKO mice, RAmKO mice showed no changes in their liver or plasma profile, indicating that the consequences of early mTORC1 inhibition are limited to the skeletal muscle.

Interestingly, even if RAmKO and TSCmKO mice initially show an opposite metabolic phenotype, both mutant mice are resistant to HFD. Both mutant mice did not gain weight nor did they develop hepatic steatosis on a 12-week-long HFD. However, while TSCmKO mice showed increased insulin sensitivity and seemed to accelerate their metabolism by eating more and being more active, RAmKO mice showed improved glucose tolerance and slowed their metabolism by decreasing the activity and their respiratory exchange ratio. All these metabolic changes, compared to those previously reported, point to the specific consequences of mTORC1 deregulation depending on the metabolic organs in which the perturbation occurs.

Consistent with the inhibition of mTORC1 activity, RAmKO mice display a downregulation of glycolytic proteins and genes involved in fatty acid oxidation in the skeletal muscle. This correlated with the increase in class II HDACs, which have been described to regulate the transcription of glycolytic proteins [8]. The inefficient nutrient utilization and the increased energy demand might lead to beneficial systemic effects and to a resistance to diet-induced obesity in the RAmKO mice. This phenotype is paralleled by a reduction of the oxidative capacity of the muscles and by a reduction of the number of mitochondria [31]. Similar to RAmKO mice, treatment with rapamycin leads to a reduced glucose uptake [3], which highlights the critical role of muscle mTORC1 signaling.

Changes in muscle integrity can affect whole-body metabolism, as seen in patients with muscular dystrophies who often develop glucose intolerance and insulin resistance [9, 34]. Both TSCmKO and RAmKO mice develop a myopathy and show a reduced body weight, which suggested that they are not able to gain lean and fat mass with age as control mice do. Interestingly, myopathic RAmKO and TSCmKO mice develop insulin resistance and show lower plasma glucose and plasma insulin concentrations. It has been proposed that whole-body insulin resistance is a consequence of lipotoxicity caused by aberrant lipid metabolism in the muscle and increased intramyocellular accumulation of ceramides and diacylglycerol [2]. Accordingly, TSCmKO and RAmKO mice could suffer from lipotoxicity as they showed increased accumulation of lipids in the skeletal muscle. This accumulation of toxic lipid intermediates could be a result of endoplasmic reticulum stress in TSCmKO mice [14, 32] and activation of inflammatory pathways in RAmKO mice [5, 25]. Nonetheless, the development of insulin resistance in both mouse models after the onset of the myopathy is well in line with the metabolic complications in muscular dystrophies. It will be interesting to see whether deregulation of mTORC1 signaling could also be at the onset of those metabolic complications in muscular dystrophies.

Our results indicate that mTORC1 is a central controller of metabolic properties of muscle tissue by affecting fatty acid and glucose metabolism, glycogen storage, and oxidative capacity. We also show that the skeletal muscle mTORC1 plays an essential role in whole-body homeostasis and energy expenditure. Our data imply that the beneficial effects of rapamycin on systemic metabolism and longevity could in part be based on inhibition of mTORC1 in the skeletal muscle. Therefore, further investigation should be conducted to determine whether mTORC1 deregulation in muscular dystrophies might be the cause of the overall changes in the whole-body metabolism.

## Conclusions

In this study, we have confirmed that alterations to mTORC1 signaling pathway in the skeletal muscle directly affect whole-body metabolism, which highlights the importance of this tissue in maintaining energy stability. Moreover, we show that a proper balance in mTORC1 signaling is essential for muscle integrity and metabolic homeostasis, as both long-term activation and inhibition originate a myopathy that mimics the main metabolic complications of dystrophic patients. Thus, muscle mTORC1 could serve as a potential target to treat those metabolic complications.

## Additional files

Additional file 1: Supplementary **Figures S1 to S5.** Figure S1. Unchanged expression of Actb. (A) Actb (encoding β-actin) expression in the muscle, liver, WAT, and BAT of control (n=5) and RAmKO mice (n=5). (B) List of antibodies used. Figure S2. Metabolism of female TSCmKO and RAmKO mice. (A) Body weight, (B) lean mass, (C) fat massand (D) plasma glucose levels of 10-week-old female TSCmKO (n=9), RAmKO (n=8) and control (Ctrl) mice (n=11). (E)–(G) Fat mass (E), lean mass (F) and plasma glucose levels (G) of male RAmKO (n=4) and control (Ctrl) mice (n=6) on a HFD. Figure S3. RAmKO mice do not show changes in other organs. (A) Glycogen amount in gastrocnemius muscle of 10-week-old TSCmKO mice (n=3). (B) Western blot analysis of liver from 10-week-old TSCmKO and control (Ctrl) mice (n=4). Mice were intraperitoneally injected with insulin (+; TSC-insulin) or not (−). Protein expression is normalized to eEF2. (C)–(D) 12-week-old RAmKO mice do not show changes in Ucp2 expression in the liver and WAT (C) or Ucp1 and Ucp2 in BAT (D) compared to control mice (n=5). (E)–(F) Liver expression of genes involved in lipid (E) and glucose (F) metabolism of RAmKO mice (n=5). Figure S4. ATP levels in 12-week-old RAmKO soleus muscle (n = 5). Figure S5. Body composition in myopathic female TSCmKO and RAmKO mice. (A) Body weight, (B) lean and (C) fat mass of 40-week-old TSCmKO (n=10), 20-week-old RAmKO (n=4) and respective control (Ctrl) littermates (n=11). (D) The kidneys of 40-week-old TSCmKO mice appear polycystic. Cysts are indicated by arrows. Additional file 2: Supplementary **Tables S1 and S2.** Table S1. List of primers used. Table S2. RAmKO blood analysis.

## Abbreviations

Akt/PKB: protein kinase B
ALTL: alanine transaminase
ASTL: aspartate transaminase
ATP: adenosine triphosphate
BAT: brown adipose tissue
FGF21: fibroblast growth factor 21
GLUT4: glucose transporter 4
GTT: glucose tolerance test
HDAC: histone deacetylase
HFD: high-fat diet
IP: intra-peritoneal
ITT: insulin tolerance test
LDH: lactate dehydrogenase
mTORC1: mammalian target of rapamycin complex 1
PPARG: peroxisome proliferator-activated receptor gamma
RAmKO: raptor muscle knock-out
SREBP1: sterol regulatory element binding protein 1
TA: tibialis anterior
TBC1D1/TBC1D4: TBC1 domain family member 1/4
TSC1: tuberous sclerosis complex 1
TSCmKO: TSC1 muscle knock-out
UCP: uncoupling protein
VCO_2_: carbon dioxide volume
VO_2_: oxygen volume
WAT: white adipose tissue
