# Supplementary material for: Alterations to mTORC1 signaling in the skeletal muscle differentially affect whole-body metabolism
Source: Skelet Muscle. 2016 Mar 21;6:13. doi: 10.1186/s13395-016-0084-8 (PMC4800774; doi:10.1186/s13395-016-0084-8)

Additional file 1: Figure S1

A

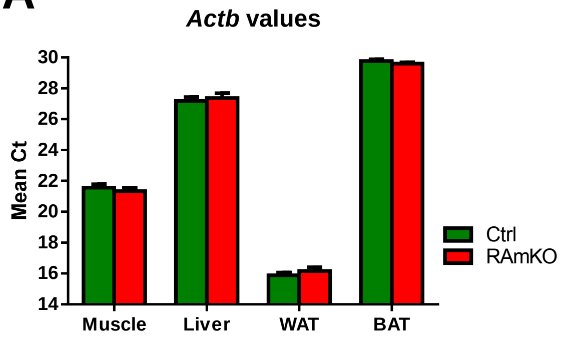

B **The following antibodies were used for immunoblotting** : β-actin (#4970), Phospho-GS Ser^{641} (#3891), Phospho-Akt Ser^{473} (#4058), Akt (#9272), Phospho-AS160 Thr^{642} (#8881), AS160 (#2670), Phospho-TBC1D1 Thr^{590} (#6927), TBC1D1 (#4629), eEF2 (#2232) and P-S6 Ser$^{235/236}$ (#2211) from Cell Signalling; α-Actinin (7732) from Sigma; G6Pase (sc-134714) and HDAC-4 (sc-11418) from Santa Cruz; UCP2 (AB3040) and HDAC-5 (#07-045) from Millipore; and UCP3 (ab3477) from Abcam.

Additional file 1: Figure S2

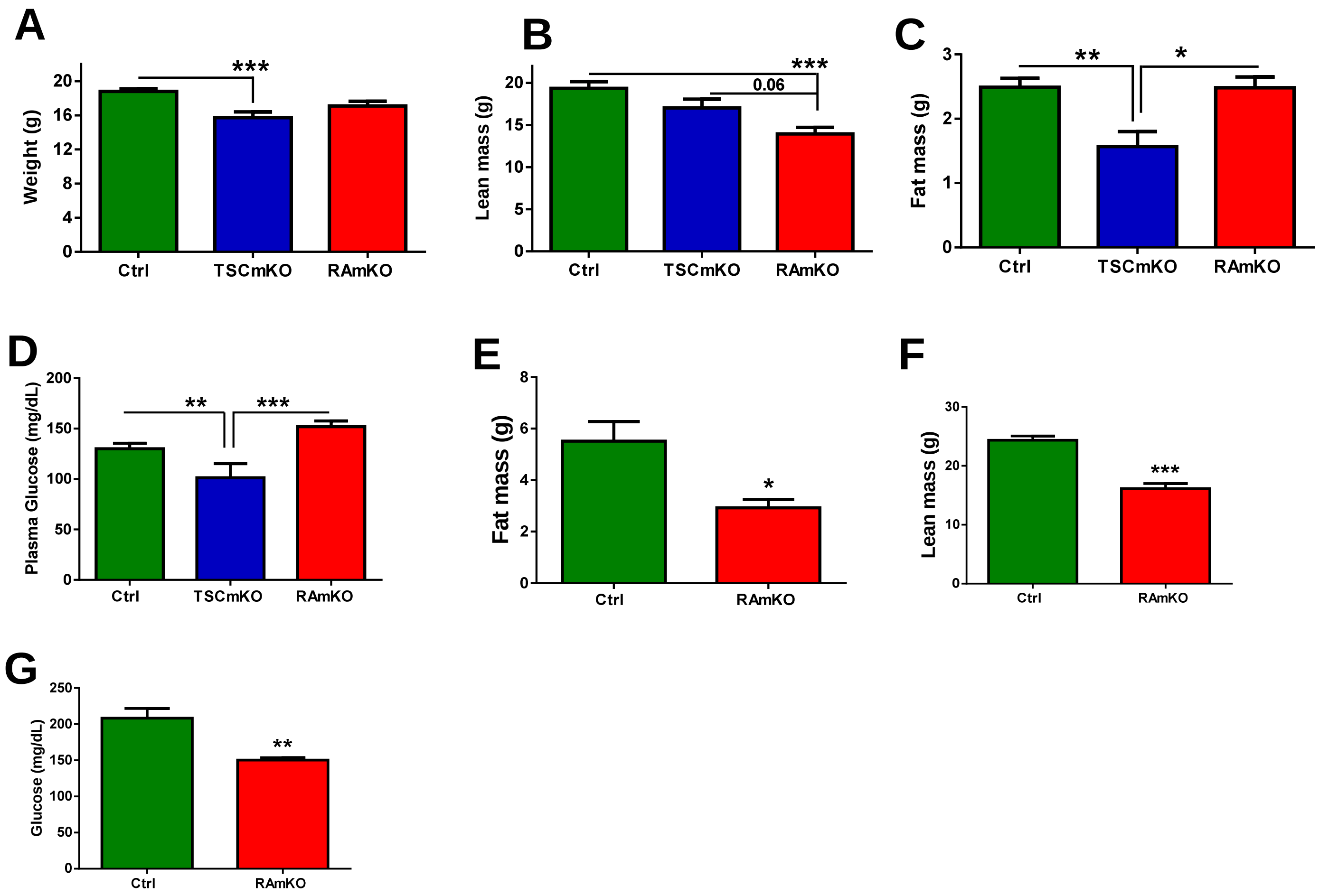

Additional file 1: Figure S3

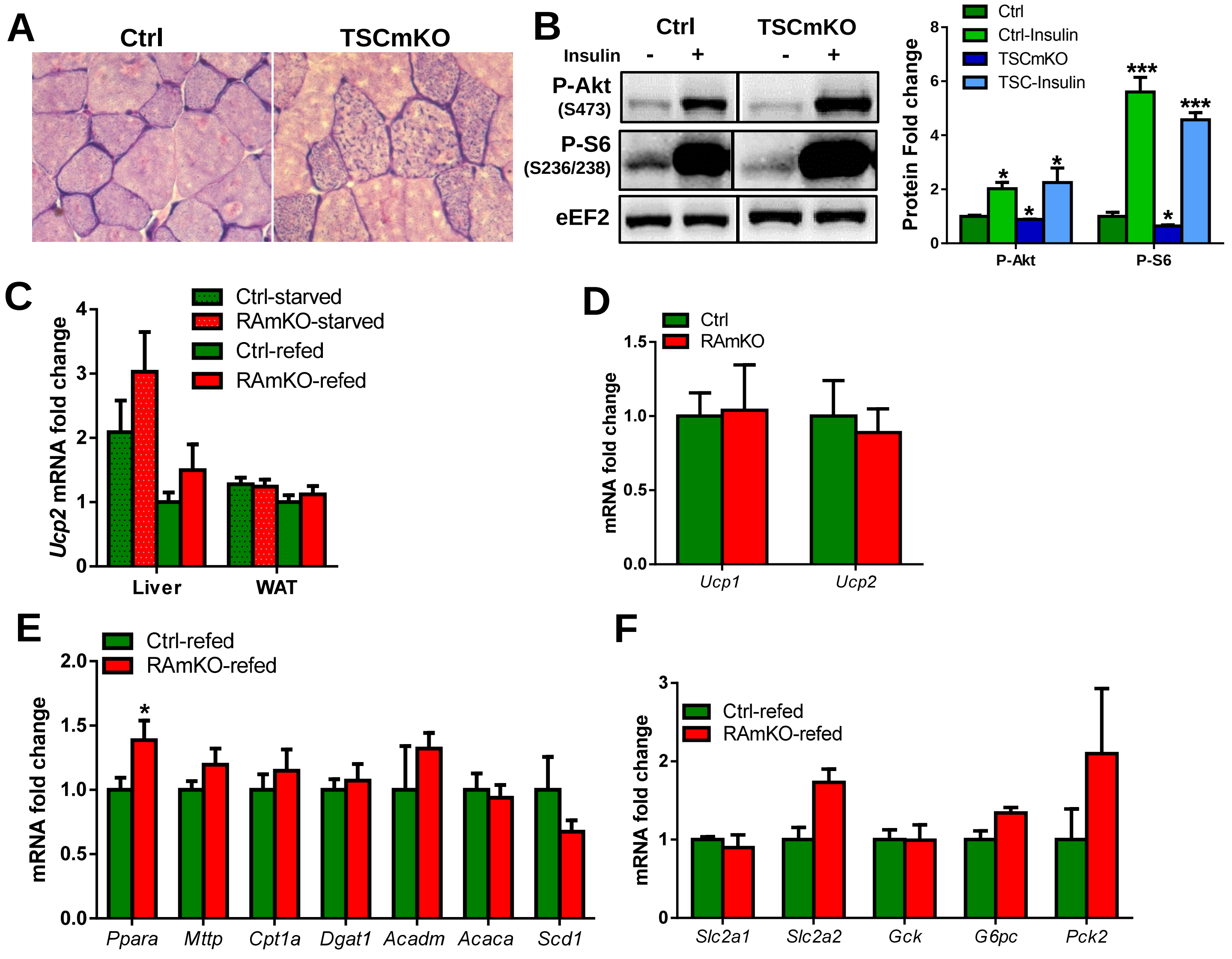

Additional file 1: Figure S4

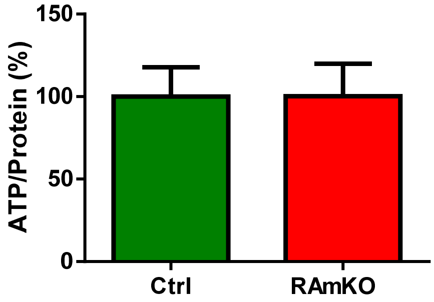

Additional file 1: Figure S5

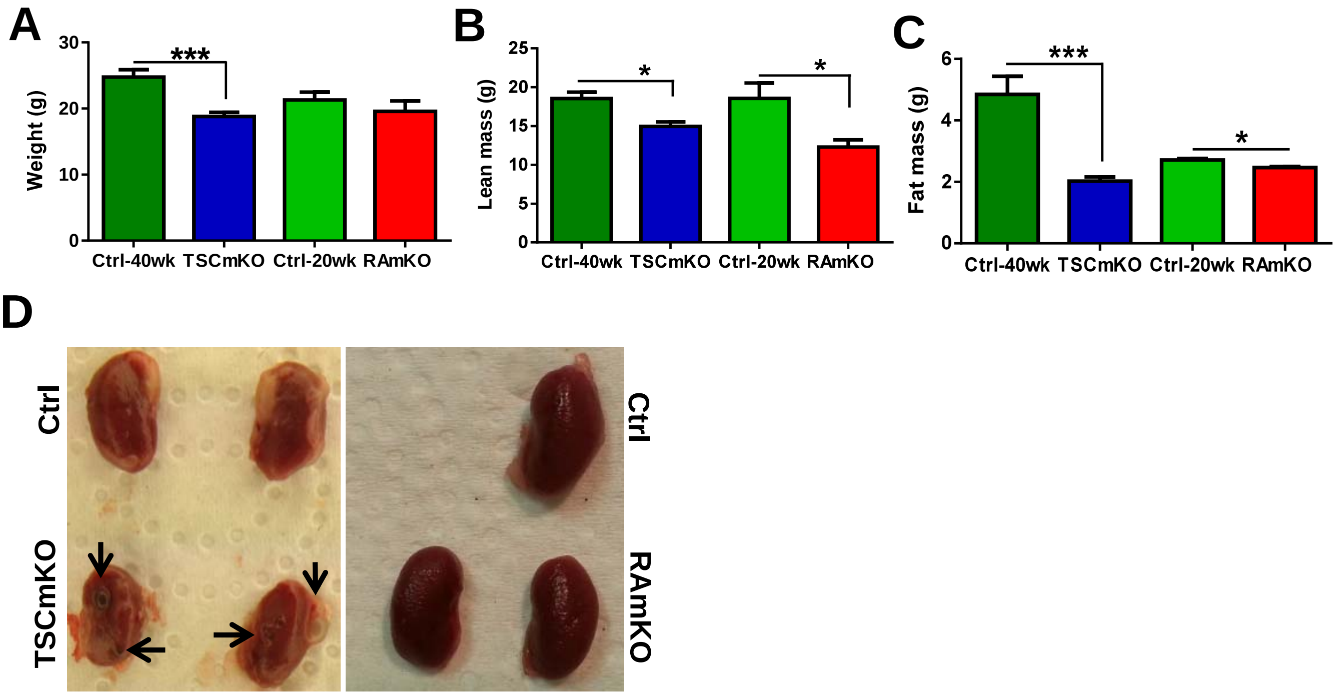

Supplement: Additional file 1: — Supplementary Figures S1 to S5. Figure S1. Unchanged expression of Actb. (A) Actb (encoding β-actin) expression in the muscle, liver, WAT, and BAT of control (n=5) and RAmKO mice (n=5). (B) List of antibodies used. Figure S2. Metabolism of female TSCmKO and RAmKO mice. (A) Body weight, (B) lean mass, (C) fat massand (D) plasma glucose levels of 10-week-old female TSCmKO (n=9), RAmKO (n=8) and control (Ctrl) mice (n=11). (E)–(G) Fat mass (E), lean mass (F) and plasma glucose levels (G) of male RAmKO (n=4) and control (Ctrl) mice (n=6) on a HFD. Figure S3. RAmKO mice do not show changes in other organs. (A) Glycogen amount in gastrocnemius muscle of 10-week-old TSCmKO mice (n=3). (B) Western blot analysis of liver from 10-week-old TSCmKO and control (Ctrl) mice (n=4). Mice were intraperitoneally injected with insulin (+; TSC-insulin) or not (−). Protein expression is normalized to eEF2. (C)–(D) 12-week-old RAmKO mice do not show changes in Ucp2 expression in the liver and WAT (C) or Ucp1 and Ucp2 in BAT (D) compared to control mice (n=5). (E)–(F) Liver expression of genes involved in lipid (E) and glucose (F) metabolism of RAmKO mice (n=5). Figure S4. ATP levels in 12-week-old RAmKO soleus muscle (n = 5). Figure S5. Body composition in myopathic female TSCmKO and RAmKO mice. (A) Body weight, (B) lean and (C) fat mass of 40-week-old TSCmKO (n=10), 20-week-old RAmKO (n=4) and respective control (Ctrl) littermates (n=11). (D) The kidneys of 40-week-old TSCmKO mice appear polycystic. Cysts are indicated by arrows. [file 13395_2016_84_MOESM1_ESM.pdf]
