# Supplementary material for: Alterations to mTORC1 signaling in the skeletal muscle differentially affect whole-body metabolism
Source: Skelet Muscle. 2016 Mar 21;6:13. doi: 10.1186/s13395-016-0084-8 (PMC4800774; doi:10.1186/s13395-016-0084-8)
Supplement: Additional file 2: — Supplementary Tables S1 and S2. Table S1. List of primers used. Table S2. RAmKO blood analysis. [file 13395_2016_84_MOESM2_ESM.docx]

**Additional file 2.**

**Table S1. Primer list.**

| *Cd36* | fw: TGGCCTTACTTGGGATTGG | bw: CCAGTGTATATGTAGGCTCATCCA |
| --- | --- | --- |
| *Scl27a1* (Fatp1) | fw: GGCTCCTGGAGCAGGAACA | bw: ACGGAAGTCCCAGAAACCA |
| *Slc27a4* (Fatp4) | fw: GGCTTCCCTGGTGTACTATGGAT | bw: ACGATGTTTCCTGCTGAGTGGTA |
| *Fabp3* | fw: CCCCTCAGCTCAGCACCA | bw: CAGAAAAATCCCAACCCAAGAAT |
| *Cpt1b* | fw: GGTCGATTGCATCCAGAGAT | bw: GACTCCGGTGGAGAAGATGA |
| *Acot2* (Mte1) | fw: TGGGAACACCATCTCCTACAA | bw: CCACGACATCCAAGAGACCA |
| *Slc2a1* (Glut1) | fw: CGAGGGACAGCCGATGTG | bw: TGCCGACCCTCTTCTTTCAT |
| *Slc2a4* (Glut4) | fw: GATGAGAAACGGAAGTTGGAGAGA | bw: GCACCACTGCGATGATCAGA |
| *Hk2* | fw: CCCTGCCACCAGACGAAA | bw: GACTTGAACCCCTTAGTCCATGA |
| *Pkm* | fw: CGATCTGTGGAGATGCTGAA | bw: AATGGGATCAGATGCAAAGC |
| *Pfkm* | fw: CAGATCAGTGCCAACATAACCAA | bw: CGGGATGCAGAGCTCATCA |
| *Ldha* | fw: TGTCTCCAGCAAAGACTACTGT | bw: GACTGTACTTGACAATGTTGGGA |
| *Ucp2* | fw: ACCAAGGGCTCAGAGCATGCA | bw: TGGCTTTCAGGAGAGTATCTTTG |
| *Ucp3* | fw: ACTCCAGCGTCGCCATCAGGATTCT | bw: TAAACAGGTGAGACTCCAGCAACTT |
| *Actb* (β-actin) | fw: CAGCTTCTTTGCAGCTCCTT | bw: GCAGCGATATCGTCATCCA |
| *Scd1* | fw: CAAGCTGGAGTACGTCTGGA | bw: CAGAGCGCTGGTCATGTAGT |
| *Mttp* | fw: CGTCCACATACAGCCTTGAC | bw: CCACCTGACTACCATGAAGC |
| *Ucp1* | fw: GGCCTCTACGACTCAGTCCA | bw: TAAGCCGGCTGAGATCTTGT |
| *Dgat1* | fw: CATGCGTGATTATTGCATCC | bw: ACAGGTTGACATCCCGGTAG |
| *Acaca* (Acc1) | fw: ACCTTACTGCCATCCCATGTG | bw: GTGCCTGATGATCGCACGAACAAA |
| *Acadm* (Mcad) | fw: TCTCGAAGACGTCAGAGTGC | bw: TGCGACTGTAGGTCTGGTTC |
| *G6Pc* | fw: AGCGGAATGGGAGCAACTTG | bw: CAGAATGGGTCCACCTTGACAC |
| *Gck* | fw: CCCTGAGTGGCTTACAGTTC | bw: ACGGATGTGAGTGTTGAAGC |
| *Pck2* (Pepck) | fw: CATCCAGGCAATGTCATCGC | bw: GCATAACTAACCCGAAGGCAAG |
| *Slc2a2* (Glut2) | fw: GTCCAGAAAGCCCCAGATACC | bw: GTGACATCCTCAGTTCCTCTTAG |
| *Ppara* | fw: TGTTTGTGGCTGCTATAATTTGC | bw: GCAACTTCTCAATGTAGCCTATGTTT |

| **Table S2. RAmKO blood analysis** | |  |  |  |  |  |  |
| --- | --- | --- | --- | --- | --- | --- | --- |
|  | 10-week-old |  |  |  | 20-week-old |  |  |
|  | Ctrl | RAmKO | P |  | Ctrl | RAmKO | P |
| pO2 [mm Hg] | 82.8 ±16.3 | 97.3 ±17.1 | ns |  | 90.7 ±7.2 | 68.7 ±5.6 | *** |
| pCO2 [mm Hg] | 18.5 ±5.8 | 16.4 ±4.0 | ns |  | 18.45 ±2.3 | 32.8 ±8.2 | ** |
| pH | 7.5 ±0.06 | 7.6 ±0.07 | ns |  | 7.5 ±0.07 | 7.4 ±0.05 | * |
|  |  |  |  |  |  |  |  |
| Values represent mean ± SEM. Student's test *p<0.05, **p<0.01, ***p<0.001 (n= 4). | | | | | | | |
